# Determination of triacylglycerol and fatty acid compositions of *Impatiens* seed oils using reverse phase high performance liquid chromatography

**DOI:** 10.55730/1300-0527.3440

**Published:** 2022-06-13

**Authors:** Anh Van NGUYEN, Anh Thi Ngoc VU, Victor Ivanovic DEINEKA, Huy-Trung-Kien NGUYEN

**Affiliations:** 1 Faculty of Food Science and Technology, Ho Chi Minh City University of Food Industry, Ho Chi Minh City, Vietnam; 2 Environmental Analysis Laboratory, Southern Branch of Vietnam-Russia Tropical Center, Ho Chi Minh City, Vietnam; 3 Institute of Engineering Technologies and Natural Sciences of Belgorod National Research University, Belgorod, Russia; 4 The American School, Ho Chi Minh City, Vietnam

**Keywords:** Conjugated fatty acid, *Impatiens* seed oil, triacylglycerols, fatty acid, *α*-parinaric acid

## Abstract

In the present work, triacylglycerol and fatty acid compositions of *Impatiens balsamina L*. and *Impatiens walleriana Hook.f*. seed oils were determined using reverse phase high performance liquid chromatography with both refractive index and spectrophotometric detections. The presence of conjugated octadecatetraenoic moieties was confirmed by UV and IR spectroscopy. Triacylglycerol (TAG) compositions were performed using an incremental approach and confirmed by the results of MS and electronic spectra. The quantitative analysis of TAG was achieved by careful calibration, introducing correction factors for the sensitivity of each compound. The results showed that both *Impatiens* seed oils contain the same 23 TAGs. The mole fraction of 15 TAGs containing conjugated moieties was more significant than 88% (for *Impatiens balsamina L*.) and 81% (for *Impatiens walleriana Hook.f*.). Seed oils of *Impatiens balsamina* and *Impatiens walleriana* contain 43.44% and 36.12% mole of conjugated octadecatetraenoic fatty acids, respectively. These conjugated fatty acids were determined to be α-parinaric (C18:4^9Z,11E,13E,15Z^) and β-parinaric (C18:4^9Z,11E,13E,15Z^), in which isomer β-parinaric represents 23.21% and 26.27% of conjugated octadecatetraenoic acids for *I. balsamina* and *I. walleriana* seed oils, respectively. In addition, the mole fraction of *α*-linolenic acid in both seed oils was also abundant at 24.5% and 28.2% for *I. balsamina* and *I. walleriana*. Therefore, *I. balsamina L*.and *I. walleriana* seed oils are potential sources of polyunsaturated fatty acids, especially conjugated octadecatetraenoic acids.

## 1. Introduction

Unsaturated fatty acids are nutritional components providing the body with specific health benefits. Both monounsaturated and polyunsaturated fatty acids (PUAF) are essential acids that the body needs to maintain brain function and cell growth and reduce the risk of cardiovascular disease [1]. In particular, conjugated fatty acids have been shown to possess many valuable biological activities, including anticarcinogenic [2], antiatherogenic, antiinflammatory [3], and antidiabetic effects [4] and immune-modulating properties [5]. Conjugated fatty acids are synthesized in nature mainly in several forms, such as conjugated linoleic acids (CLAs), conjugated linolenic acids (CLnAs), and conjugated stearidonic acids (CSAs). CLAs usually exist mainly in dairy products with tiny concentrations, making them difficult to extract and commercialize, while CLnAs and CSAs were found as major components in some seed oils [6]. Conjugated stearidonic acid has been biosynthesized from stearidonic acid via microbial enzyme activity [7]. α-parinaric (C18:4^9Z,11E,13E,15Z^) and acid β-parinaric (C18:4^9E,11E,13E,15E^) –a pair of CSA were found in some plant seed oils and algae. CSAs, like the other conjugated fatty acids, showed anticarcinogenic activity in vitro, including monocytic SW480 colon cancer cells, leukaemia cells, and malignant gliomas [8–10].

Analysis of the TAG composition plays an essential role in evaluating the quality of seed oils because of several advantages. Firstly, analyzing TAG compositions provides information on fatty acid composition in the oil without transferring TAGs to methyl esters. The conversion of some PUFAs to fatty acid methyl esters could cause changes in the fatty acid compositions, such as their isomerization, oxidation, and decomposition. According to the literature results [11,12], conjugated fatty acids were isomerized in acidic and basic reagents during the transformation of TAGs to methyl esters. In addition, the analysis of TAG compositions provides information on the moieties distribution in a series of TAGs, an important feature that can be used in the falsification analysis of fat samples [13,14]. Reverse phase high performance liquid chromatography (RP HPLC) was demonstrated to be suitable for analyzing TAG compositions of seed oils, especially for TAG containing chemically highly labile conjugated unsaturated acid substituents [11,15].

*Impatiens L*. is one of the enormous genera of seed plants of flowering plants in the family Balsaminaceae containing more than 1000 species [16]. The seeds of some *Impatiens* species have high oil yields, a principal constituent of which is unusual conjugated fatty acid as known parinaric acid [17, 8]. The *Impatiens balsamina L*. and *Impatiens walleriana Hook.f*. are species of horticultural value and widely cultivated in many regions. α-parinaric acid was found in the seed oil of *I. balsamina* using ^13^C nuclear magnetic resonance spectroscopic analysis [18] and gas chromatography [19]. However, the information on the TAG and fatty acid compositions of the seed oils of the two species has lacked. This study aims to determine the qualitative and quantitative compositions of triacylglycerols and fatty acids of *Impatiens balsamina L*. and *Impatiens walleriana Hook.f*. using RP HPLC.

## 2. Material and method

### 2.1. Reagents and sample collection

Seeds of *Impatiens balsamina L*. and *Impatiens walleriana Hook.f*. were collected in Lao Cai, Vietnam, in 2020. Linseed oil was purchased through the Aromarti.ru online store. All seed samples were stored in brown glass bottles until analysis.

HPLC grade acetonitrile, acetone, and propan-2-ol were obtained from Sigma-Aldrich. Other reagents for extraction and purification were all reagents grade without further purification.

### 2.2. Oil extraction and purification

The seed oil of *Impatiens balsamina L*. and *Impatiens walleriana Hook.f*. (2.0 g) were extracted with 10 mL of *n*-hexane by grinding in a porcelain mortar at room temperature. The process was repeated 8 times, and the combined portions were filtered through a paper filter, and the solvent was evaporated to constant mass. Oil contents of *Impatiens balsamina L*. and *Impatiens walleriana Hook.f*. seeds were determined to be 27.3 ± 0.2% and 23.5 ± 0.3% (n = 3), refractive index $n_{D}^{25}=1.5011$ and 1.4995, respectively.

Extracted oils were purified by solid-phase extraction on DIAPAK C cartridges (BioChemMak ST, Moscow) using silica gel for the stationary phase. For the purification [20], 200 mg of seed oils were dissolved in 20 mL n-hexane, and the solution was passed through the cartridge. The desorption was performed by 4 mL of acetone.

### 2.3. Chromatographic conditions

For the separation of TAGs, Shimadzu LC20 chromatographic system with refractive index detector (RID 10A) and an Agilent 1200 Infinity chromatograph system with diode array and MS detectors were used. Chromatograms were recorded using mobile phases of the systems “acetonitrile-propan-2-ol” and “acetone – acetonitrile” for refractive index (RI) and DAD detector, respectively. The speed of the mobile phase was 0.8 mL/min; chromatographic columns of 250 × 4.6 mm Kromasil 100-5C18 (for HPLC with spectrophotometric detection) and 150 × 2.1 mm Kromasil 100–5C18 (for MS detection) were used at a thermostat temperature of 30°C.

Mass spectrometric detection (6130 Quadrupole MS, Agilent) was carried out in the atmospheric pressure chemical ionization mode under standard conditions at a fragmentor voltage of 150 V; signals were recorded for positively charged ions. The Kromasil 110-3.5C18 2.1 × 150 mm column was used, the mobile phase speed was 0.1–0.2 mL/min, and the eluent system was acetonitrile– propanol-2 with additions of ammonium formate (HCOONH_4_) 0.2 mM. All the chromatograms were performed in isocratic mode. The MagicPlot Student software was used for the resolution of “problem” (with a low value of R_S_) TAG.

### 2.4. Qualitative and quantitative analysis of TAG

The TAG compositions of two *Impatiens* seed oils were performed by the incremental approach [21, 22] based on the retention parameters, and the compositions of TAG were confirmed using the information on both their molecular ions of MS and electronic absorption spectra. The capacity factors (k) were deduced using column void time (t_o_), calculated by the homologous series method [23]. In the present work, the retention time of series Pr_3_ - Pr_2_Ln - PrLn_2_ - Ln_3_ was used, and void time (t_o_) was calculated to be 2.625 min and 2.358 min for Shimadzu LC20 chromatographic and Agilent 1200 Infinity chromatography systems, respectively in current conditions.

Mole fraction of TAGs on the chromatogram was performed by the formula:


$$\alpha \left({TAG}_{i}\right)=\frac{\frac{S_{i}}{k_{i}}}{\sum_{i}\frac{S_{i}}{k_{i}}}\times 100,$$

where S_i_ – corresponding peak area,
α(TAG_i_) – mole fraction of TAG_i_,
k_i_ – the correction coefficient for *i*-peak; for UV detector, k_i_ – the number of conjugated octadecatetraenoic acid substituents in TAG; and for RI detector, the correction coefficients were taken into account in the calculations, which take into account the change in the response of the refractometric detector with a change in the TAG composition, proposed in [24].

Mole fraction of fatty acid was calculated using mole fractions of all TAGs, taking into account the numbers of the acid substituents (n_ij_), in each TAGs:


$$\alpha \left({Acid}_{j}\right)=\frac{\sum_{i}\alpha \left({TAG}_{i}\right)\times n_{ij}}{\sum_{j}\sum_{i}\alpha \left({TAG}_{i}\right)\times n_{ij}}.$$

### 2.5. Spectrophotometric measurement

The electronic absorption spectra of *Impatiens balsamina L*. and *Impatiens walleriana Hook.f*. n-hexane seed oils extract were performed by quartz cells (1 cm) on a LAMBDA 365 UV/Vis Spectrophotometer.

The FTIR spectra of clean oils were recorded by *Spectrum Two FT-IR* Spectrometer from 400 cm^−1^ to 4000 cm^−1^.

### 2.6. Abbreviation of triacylglycerols and fatty acid

TAGs were abbreviated by letters representing fatty acid moieties without specification of their position in the molecule: α-Pr represents the radical of C18:4^9Z,11E,13E,15Z^ (α-parinaric acid); α-Ln-C18:3 ^9Z,12Z,15Z^ (linolenic acid); L-C18:3 ^9Z,12Z^ (linoleic acid); O - C18:1^9Z^ (oleic acid); P - C16:1 (palmitic acid); and S - C18:0 (stearic acid). For example, the formula, Ln_2_L represents a TAG with two substituents of linolenic acid and one of linoleic acid.

All results of TAG and fatty acids compositions were performed by triplicate measurements and expressed as mean ± standard deviations.

## 3. Results and discussion

### 3.1. Confirmation of the presence of conjugated fatty acid moieties

**Figure 1:**
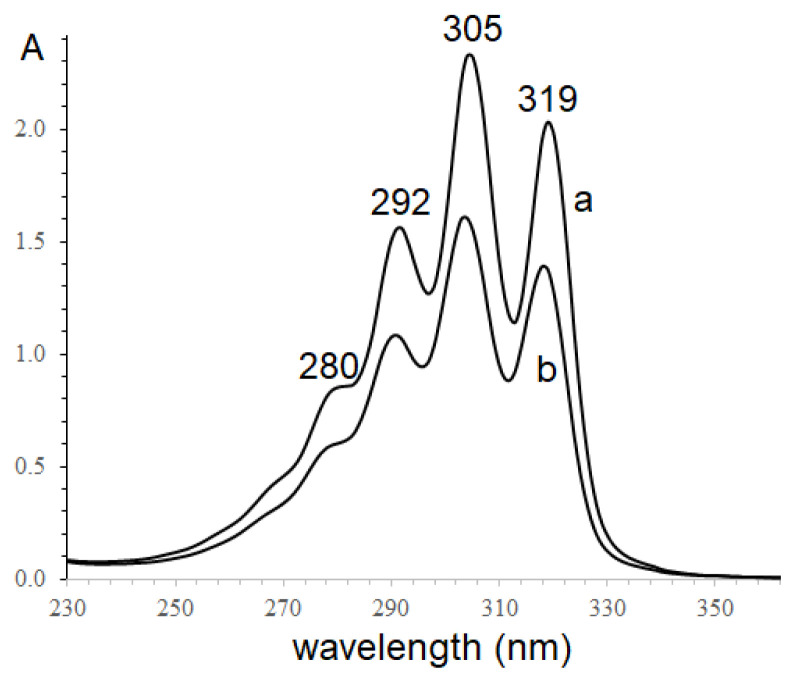
Ultraviolet absorption of n-hexane extract seed oils; a- *Impatiens balsamina*; b- *Impatiens walleriana*.

According to the presence of α-parinaric in some *Impatiens* species seed oils, in this study, conjugated octadecatetraenoic acids in the seed oils of *Impatiens balsamina* and *Impatiens walleriana* were confirmed using UV and IR spectroscopy due to the specificity of conjugated fatty acid spectra. The *n*-hexane extracts of two seed oils were recorded and shown in Figure 1.

**Figure 2:**
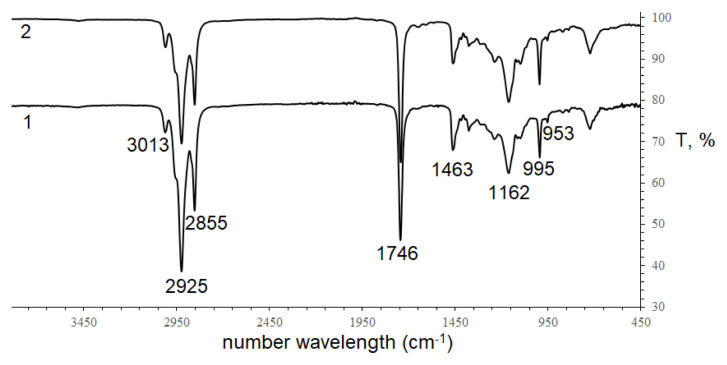
FTIR spectra of two *Impatiens* seed oils 1- *Impatiens balsamina*; 2- *Impatiens walleriana*.

The ultraviolet spectra of two *Impatiens* seed oils were observed with the absorption maxima at 293, 305, and 319 nm, characteristic of conjugated tetraenoic compounds [10–11]. The maxima absorption of *I. walleriana* seed oil slightly shifts to a shorter wavelength, indicating the different content of conjugated tetraenoic acid isomers. In addition, conjugated fatty acid exhibits different selective absorption in the 950–1000 cm^−1^ region of the infrared spectrum, in which IR spectra of conjugated octadecatetraenoic fatty acid are not unlike spectra of conjugated trienoic acids [27]. In this work, the FTIR spectra of two *Impatiens* seed oils were studied and shown in Figure 2.

Both infrared spectra of these seed oils have typical features of triacylglycerols, containing high-level unsaturated fatty acids. The 2800–3020 cm^−1^ region was observed with strong bands due to the C-H stretching absorptions of the -CH_2_- and CH_3_- groups of the fatty acid moieties and the C-H stretching absorption of -CH=CH- at 2855 cm^−1^; Around 1750 cm^−1^, strong stretching absorption (C=O) was observed. Moreover, IR spectra of two seed oils have two absorption bands in the 950–1000 cm^−1^ region (995 and 953 cm^−1^), assigned to conjugated C=C [27]. These UV and FTIR spectra results confirmed the presence of conjugated tetraenoic moieties in the oils of *I. balsamina* and *I. walleriana* seed.

### 3.2. Determination of TAG components of Impatiens seed oils

Due to the presence of conjugated tetraenoic moieties in seed oils of two *Impatiens* species, TAGs can be recorded on the chromatogram using a spectrophotometric detector, and a diode-array detector is a powerful tool in peak identification and confirmatory analysis. However, before using spectrophotometric detection, it is necessary to ensure the absence or estimate the proportion of TAGs that do not contain conjugated acid radicals using refractometric detection. For this purpose, TAGs of *Impatiens* seed oils were separated using isocratic elution mode with a mobile phase of acetonitrile and acetone.

**Figure 3:**
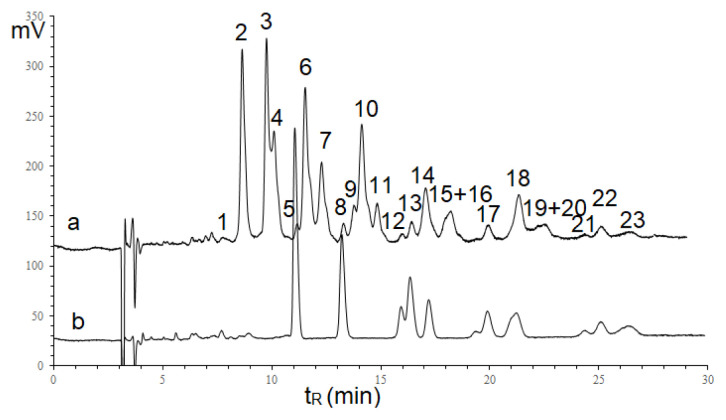
Chromatograms of *Impatiens walleriana* and Linseed oils Column 4.6× 250 mm Kromasil 100× 5C18, mobile phase compositions: acetonitrile: acetone (3:7, v/v), 0.8 mL/min, refractometric index detector, peak numbers seen in Table 1.

Because of the existence of α-linolenic acid in seed oils of some *Impatiens* species [19], the chromatogram of *I. walleriana* seed oil was recorded under the background of the linseed oil chromatogram using RI detector and shown in Figure 3.

On the chromatogram (Figure 3A), 23 TAGs were separated and determined using the incremental approach [21]. The results of processing the peak retention parameters and TAG components are presented in Table 1. The results showed that the same changes in TAG structures correspond to the same increments - for replacing linolenic acid (Ln) with linoleic acid (L) (0.103 logarithmic units); linoleic acid (L) with oleic acid (O) (0.118 logarithmic units), oleic acid (O) with palmitic acid (P) (0.027), and palmitic acid with stearic acid (0.112). At the same time, moiety X of *I. walleriana* seed oil, significantly eluted earlier than linolenic moiety, indicated that moiety X contains more than three C=C double bonds. According to the UV and IR spectra results above, moiety X should be a conjugated octadecatetraenoic substituent. As shown in Figure 3, the separation of 23 TAGs was performed by current chromatography condition. However, two TAG pairs, XLP+XLnS and XLS+XOP were not completely separated; their retention time and peak area were handled by Magicplot Student software, representing individual components by unmodified Gaussians (Figure 1S in Supplementary Information).

**Figure 4:**
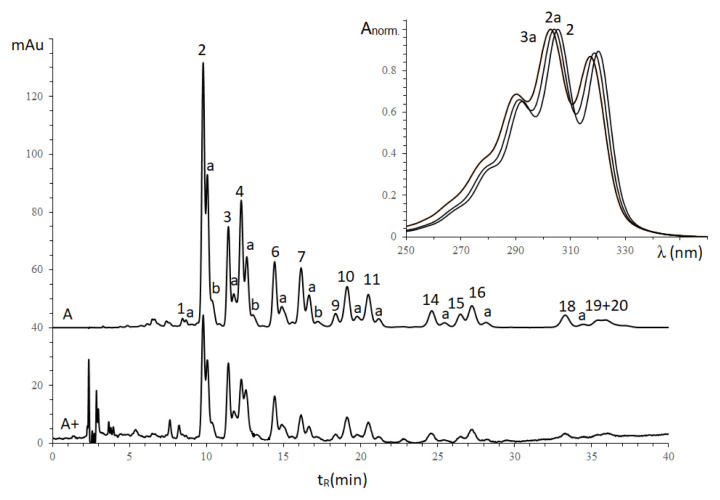
Chromatograms of *I. walleriana* seed oils. DAD detector, mobile phase: 35% propan-2-ol in acetonitrile; speed 0.8 mL/min; Chromatogram A was registered at λ=304nm; A+: at λ=210nm (with 100 times increasing of the intensive); ‘a’ and ‘b’ mark TAGs in which one and two α-parinaric moieties were replaced by β-parinaric moieties, respectively.

As shown in Figure 3, conjugated octadecatetraenoic isomers in seed oils could not be determined using a RI detector due to the separation problems. However, based on the electronic absorbance specific to conjugated double bond moieties, the aspect of determining the configuration of conjugated tetraenoic acid was discussed using diode-array and MS detectors. The separation of TAG components with the mobile phase of propan-2-ol and acetonitrile at two wavelengths was shown in Figure 4.

In the case of detection at 304 nm **(**Figure 4A), only the peaks of TAGs containing the conjugated octadecatetraenoic moieties were visible; those with nonconjugated acid substituents cannot be detected directly. Thus, the proportion of TAGs according to the peak areas at 304 nm was somewhat different from that for refractometric detection. Indeed, the area ratio of pair X_2_Ln and XLn_2_ has a remarkable change when comparing two chromatograms recorded by different detectors. In addition, detection at 210 nm allows visibility of TAGs containing either linolenic or octadecatetraenoic acid substituents, although the sensitivity towards conjugated tetraenoic compounds decreases significantly compared with the detection at 304 nm. As a result, the proportion of TAG peak areas at 304 nm and 210 nm is significantly different. This information also confirmed the simultaneous presence of conjugated octadecatetraenoic and linolenic moieties in the seed oil.

Based on the retention time parameters of separated peaks, the TAG components were worked out using an incremental approach and listed in Table 2.

The results of TAG compositions determined using an incremental approach were also confirmed by the mass spectra parameters obtained for the molecular ion [M+H]^+^ and electronic absorption spectra (Table 2**)**. The TAGs of the 20 peaks (numbered in Figure 4) have undifferentiated electronic absorption spectra with three maximum absorbances at 293, 305, and 320 nm, indicating the presence of conjugated octadecatetraenoic moieties as known α-parinaric (C18:4^9Z,11E,13E,15Z^) in these TAGs. Moreover, there are “duplicates” of the main peaks with slightly increased retention times (marked with the letters “a” and “b”). These electron absorbances of “duplicates” peaks are characterized by a slightly hypochromic shift of the absorption band, and these peaks have identical mass spectra indicating the replacement of one moiety from trans- to cis- configuration. For example, three peaks No 2, 2a, and 2b in Figure 4 had identical MS spectra with the molecular ion of 869.5 ([M+H]^+^) and electron absorbances with the maximum at 305, 304, and 302.5 nm, respectively. Therefore, these peaks No 2, 2a, and 2b were assigned as αPr_2_Ln, αPrβPrLn, and βPr_2_Ln.

**Figure 5:**
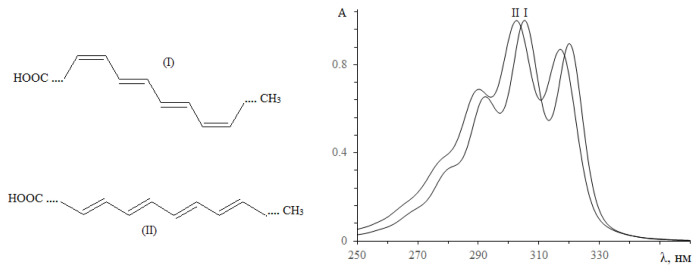
Electronic spectra of TAG PrL_2_ Radical Pr: I - α-parinaric and II - β-parinaric acids. Solvent: 35:65% v/v propan-2-ol and CH_3_CN.

A distinctive feature of the electronic spectra of conjugated polyene compounds is their electronic-vibrational structure [28], the bands which correspond to electron transitions from the ground vibrational state of the ground electronic state into several different vibrational states of the first excited electronic state due to the “verticality” (i.e. delay in the change in the nuclear configuration when the electronic configuration changes). Following the empirical rules, a hypsochromic shift of the absorption maxima should be observed when the trans-configuration replaces the cis-configuration. In the case of octadecatetraenoic acids, the electronic spectra recorded in the detector cuvette (as shown in Figure 5) have an electronic-vibrational structure characteristic of conjugated tetraene compounds with three maxima absorbent at 293, 305, and 320 nm (for α-parinaric acid) while for β-parinaric moiety with all trans-conformation at 290, 302 and 317 nm.

According to the literature data, both the stereochemistry of the double bond and the number of double bonds in the conjugated system affected the position of maximum electronic absorbance. There is an approximate dependence between the wavelength of the absorption maximum and the length of the conjugation chain. According to the literature data [29], conjugation causes a bathochromic shift of the absorption band, proportional to the number of double bonds C=C of the conjugated system in the chromophore of the molecule. Between the squares of absorption maxima wavelengths and the number of double bonds in a conjugated system for a similar configuration of these C=C bonds had the dependence:


$${\lambda_{\text{Max}}}^{2}=\text{A}+\text{B}\times \text{n},$$

where n is the number of double bonds in conjugation in the chromophore of the molecule.

**Figure 6:**
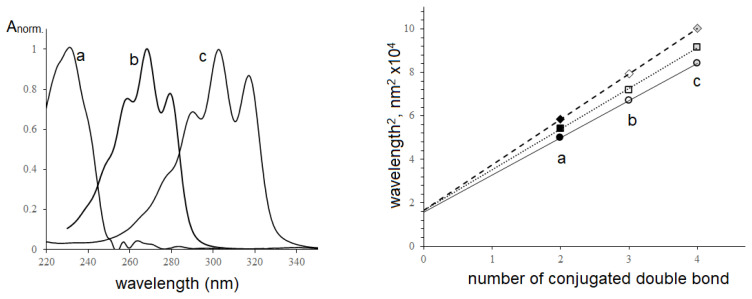
Wavelength squared of the absorption maxima for various conjugated moieties vs. the number of double bonds in the conjugated system. a- electronic spectra of 10E,12E-octadeca-10,12-dienoic acid; b- electronic spectra of 9E,11E,13E-octadeca-9,11,13-trienoic acid C18:3^9E,11E,13E^; c- electronic spectra of 9E,11E,13E,15E-octadeca-9,11,13,15-trienoic acid C18:4^9E,11E,13E,15E^.

In the present work, this relationship was confirmed using the spectra of the number of conjugated moieties with complete trans configuration: tetraenoic (β-parinaric acid C18:4^9E,11E,13E,15E^) (present in this work), trienoic (β-eleostearic acid C18:3^9E,11E,13E^) [15] and dienoic (C18:2^9E,11E^) [30] (as shown in Figure 6). There are three cusps of electronic spectra of pure conjugated trienoic and tetraenoic moieties, while these three cusps of conjugated dienoic acid were overlapped and carefully separated by the unmodified Gaussians function.

According to the results of fatty acid composition of *I. Balsamina* determined by GC-FID [19], the molar fraction of parinaric acid slightly exceeds 27%. In the present work, TAG compositions of this oil were determined using both DAD and RID detectors.

**Figure 7:**
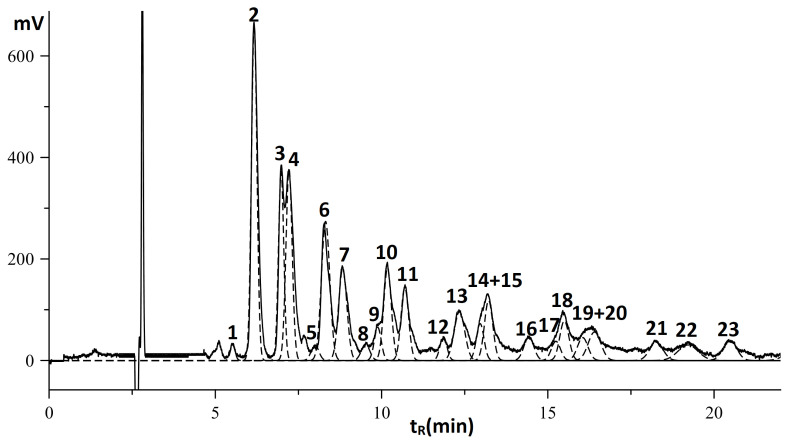
Chromatogram of *Impatiens balsamina* seed oils Column 4.6× 250 mm Kromasil 100× 5C18, mobile phase compositions: acetonitrile: acetone (5:13, v/v), 0.8 mL/min, refractometric index detector; TAG composition: 1. Pr_3_; 2. Pr_2_Ln; 3. PrLn_2_; 4. Pr_2_L; 5. Ln_3_; 6. PrLnL; 7. Pr_2_O; 8. Ln_2_L; 9. PrL_2_; 10. PrLnO; 11. PrLnP; 12. LnL_2;_ 13. Ln_2_O; 14. PrLO; 15. PrLP; 16. PrLnS; 17. LnLO; 18. PrO_2_; 19. PrLS; 20. PrOP; 21. L_2_O; 22. LnO_2_; 23. LnOP. The chromatogram was processed by MagicPlot Student.

**Figure 8:**
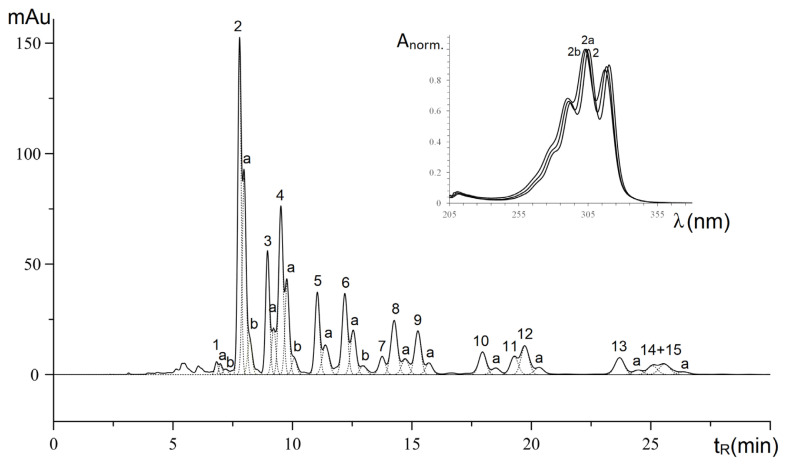
Chromatogram of *I. walleriana* seed oils; DAD detector at 306 nm, mobile phase: 35% propan-2-ol in acetonitrile ; 0.8 mL/min; TAG composition: 1-αPr_3_; 2- αPr_2_Ln; 3- αPrLn_2_; 4- αPr_2_L; 5- αPrLnL; 6- αPr_2_O; 7- αPrL_2_; 8- αPrLnO; 9- αPrLnP; 10- αPrLO; 11- αPrLP; 12-αPrLnS; 13- αPrO_2_; 14- αPrLS; 15- αPrOP. ‘a’ and ‘b’ mark TAGs in which one and two α- parinaric moieties were replaced by β- parinaric moieties, respectively.

The chromatograms of *I. Balsamina* seed oils for DAD and RID detectors, processed by MagicPlot Student with the representation of individual components by unmodified Gaussians, were shown in Figures 7 and 8. The calculated TAG composition was obtained using the incremental approach and listed in Figures 7 and 8. The calculation and retention parameters of TAGs separation were shown in Supplementary Information (Table S1 and S2).

According to the presented data, TAG compositions of *I. Balsamina* and *I. Wavariana* were almost the same. On the chromatogram of *I. balsamina* of both RID and DAD detectors, the most insensitive peak was assigned for di-α-parinaric-linolenoate, significantly different from *I. walleriana* seed oil.

On the chromatogram, there were also ‘duplicate’ peaks (marked by peaks ‘a’ and ‘b’) accomplished the prominent peaks with hypochromic electron spectra and the same mass spectra, indicating the replacement of one α-parinaric by β-parinaric radical. Indeed, peaks marked as ‘a’ and ‘b’ indicated that β-parinaric radicals replaced one and two α-parinaric, respectively.

### 3.3. Determination of the quantitative TAGs and fatty acid composition

For the spectrophotometric detector, TAG quantitative composition with different chromophores could be obtained by considering that the peak areas are directly proportional to TAG mole fractions and the number of chromophores radicals at isosbestic wavelength [31]. Due to the simultaneous presence of α and β-Parinaric moieties, isosbestic wavelength may be calculated according to the method in our works [30]. The electronic absorbance spectra of three TAGs, named αPrLn_2_ (peak 3 in Figure 4), βPrLn_2_ (peak 3a in Figure 4), and αPrβPrL (peak 4a in Figure 4), were used. The normalization of the peak 3 and peak 3a spectrum gives functions F_αPr(λ)_, F_βPr(λ)_ of pure α-parinaric and β-parinaric acid derivatives, respectively. A similar procedure for the electronic spectra of αPrβPrL obtained F_αPrβPr(λ)(exp.)_. Besides, the function F_αPrβPr(λ)_ could be achieved by normalization of the sum of the two functions, taken with the sensitivity coefficient, g, at the experimental λmax value:


$${\text{F}}_{\alpha \text{Pr}\beta \text{Pr}}(\lambda )=\frac{{\text{F}}_{\alpha \text{Pr}}(\lambda )+\text{g}\times {\text{F}}_{\beta \text{Pr}}(\lambda )}{{\text{F}}_{\alpha \text{Pr}}\left(\lambda_{\text{max}}\right)+\text{g}\times {\text{F}}_{\beta \text{Pr}}\left(\lambda_{\text{max}}\right)}={\text{F}}_{\alpha \text{Pr}\beta {\text{Pr}}_{\left(\text{exp}.\right)}}(\lambda ).$$

**Figure 9:**
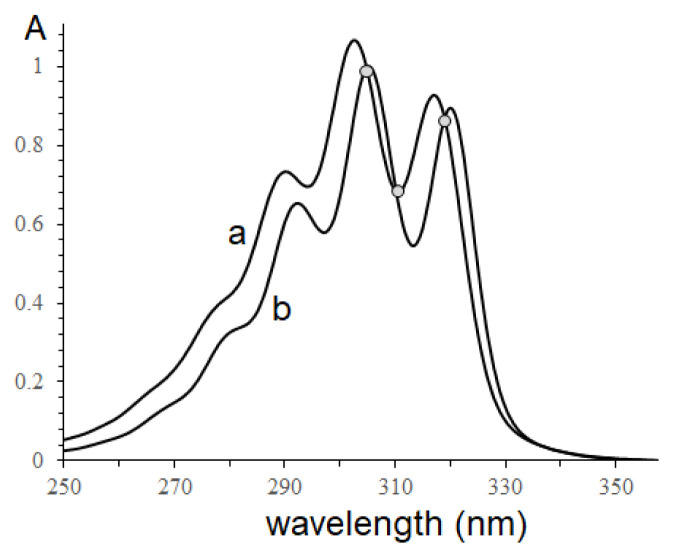
Comparison of the spectra of TAGs containing α-parinaric and β-parinaric moities with the correction factor. The ratio of extinction coefficients for α and β-parinaric radicals is determined to be g = 1.065 ± 0.004, and the isosbestic points are at 306, 310, and 319 nm.

The coefficient “g” was determined based on the mean least-square deviation between the calculated and experimental spectra in which the summation covered all wavelengths from 230 to 320 nm. Several different sets of spectra registered in several mobile phase compositions were used to calculate the coefficient g value, 1.065 ± 0.004. The plotting normalized spectrum F_αPr_(λ) and the spectrum F_βPr_(λ)×g on the same graph allows finding the isosbestic point in the place of junction of the two spectra. Three isosbestic wavelengths were 306, 310, and 319 nm (as shown in Figure 9).

The aspect of quantitative TAGs composition consists of using UV detector not allowing the detection all TAGs, not containing conjugated moieties, and the using RF detector not allowing to separate all isomers of conjugated moieties. Therefore, in the present work, we combine the results of UV and RF detectors for quantitating TAGs compositions of two seed oils. For the UV detector, the chromatograms of two species of *Impatiens* seed oils were registered at isosbestic points (306 nm), and the peak areas were used to determine mole fractions of TAGs without introducing correction factors to the sensitivity of each TAG. For RI detector, the sensitivity of the detector with various TAGs was estimated by the sensitivity value calculated using the difference in the refractive indices of TAGs, obtained by *ChemSketch* program and the refractive index of the given composition of the mobile phase [24]. The results of calculating the mole fraction of TAG compositions for *Impatiens balsamina L*. and *Impatiens walleriana Hook.f*. seed oils by refractometric and spectrophotometric detections were presented in Table 3.

As presented in Table 3, the mole fraction of TAGs, not containing conjugated tetraenoic moieties was less than 12% (for *I. balsamina*) and 19% (for *I. walleriana*). For both *Impatients* seed oils, the most dominants TAGs (mole fraction is greater than 6%) were Pr_2_Ln, PrLn_2_, Pr_2_L, PrLnL, Pr_2_O, and PrLnO. Based on the mole fraction of TAGs, the fatty acid composition of two seed oil was determined and listed in Table 4.

Both two seed oils are abundant sources of polyunsaturated fatty acids, especially the mole fraction of conjugated octadecatetraenoic and linolenic acids were more than 36 and 24%, respectively. The lack of visible nonconjugated moieties in UV detector could explain the significant difference in the mole fraction of fatty acids for the seed oil between 2 detectors. The conjugated octadecatetraenoic in these seed oils was parinaric acids, in which the main isomer was α-parinaric. Based on the UV detector results, mole fraction β-parinaric acid was calculated at 23.21% and 26.27% of conjugated octadecatetraenoic acids for *I. balsamina* and *I. walleriana*, respectively. The fatty acid composition of *I. balsamina* seed oil in this work was consistent with the results determined by GC-FID in the literature [19].

## 4. Conclusion

The present work confirmed the presence of conjugated octadecatetraenoic moieties in two species *Impatiens* seed oils using UV and IR spectroscopy. The compositions of triacylglycerols and fatty acids of *I. balsamina* and *I. walleriana* seed oils were determined by reversed-phase high pressure chromatography using both UV and RI detectors using incremental approach and confirmed by MS and electronic spectra parameters. For both seed oils, the chromatogram of 23 TAGs in RI detector was achieved with the mobile phase of acetone and acetonitrile, while 15 TAGs containing conjugated moieties were separated and determined using mobile phase of acetonitrile and propan-2-ol. The results showed that the mole fraction of TAGs, not containing conjugated tetraenoic moieties was less than 12% (for *I. balsamina*) and 19% (for *I. walleriana*). For two *Impatiens* seed oils, the most abundant TAGs (more than 6% mole) were Pr_2_Ln, PrLn_2_, Pr_2_L, PrLnL, Pr_2_O, and PrLnO. Based on the results of fatty acid compositions using two detectors, *I. balsamina* seed oil contains 43.45% parinaric acid (including 33.37% α-parinaric and 10.08% β-parinaric acid), 24.5% linolenic acid, 13.59% linoleic, 11.94% oleic acid, and 6.51% unsaturated fatty acid substituents, while for *I. walleriana* seed oil contains 27.07%, 9.65%, 28.20%, 14.00%, 15.87% and 5.18% of α-parinaric, β-parinaric, linolenic, linoleic, oleic and saturated fatty acid, respectively. Therefore, these *Impatiens* seeds are a valuable potential source of oils containing the enrichment of biologically active substances.

## Electronic supporting information

Figure 1S Chromatograms processed by MagicPlot Student of *Impatiens walleriana*; Column 4.6× 250 mm Kromasil 100× 5C18, mobile phase compositions: acetonitrile: acetone (3:7, v/v), 0.8 mL/min, refractometric index detector.

**Table S1:** Chromatography parameters and TAG components of *I. balsamina* seed oil.

No	TAGs	t _R_(min)	logk	Increments Δ(j→i) (± 0.002)				
				X_Ln	Ln_L	L_O	O_P	P_S
1	X _3_ *	5.500	0.038					
2	X _2_Ln	6.157	0.127	0.090				
3	XLn _2_	6.983	0.219	0.091				
4	X _2_L	7.218	0.242		0.114			
5	Ln _3_	7.980	0.308	0.090				
6	XLnL	8.272	0.331		0.113			
7	X _2_O	8.816	0.371			0.130		
8	Ln _2_L	9.570	0.421	0.090				
9	XL _2_	9.930	0.443		0.112			
10	XLnO	10.220	0.460	0.089		0.129		
11	XLnP	10.710	0.487				0.027	
12	LnL _2_	11.950	0.549	0.089		0.128		
13	Ln _2_O	12.430	0.571			0.128		
14	XLO	13.030	0.597				0.026	
15	XLP	13.200	0.604					0.117
16	XLnS	14.690	0.661	0.090	0.112			
17	LnLO	15.440	0.688				0.026	
18	XO _2_	15.750	0.698			0.127		
19	XLS	16.290	0.715					0.118
20	XOP	16.530	0.723			0.126		
21	L _2_O	18.220	0.773		0.111			
22	LnO _2_	18.750	0.787	0.089				
23	LnOP	20.010	0.820	0.097			0.033	
Mean value				0.083	0.103	0.118	0.027	0.112

* X- conjugated octadecatetraenoic moiety.

**Table S2:** Chromatography and mass spectra parameters and TAG components of seed oils *I. balsamina*.

No *	TAGs	t _R_(min)	logk	Increments Δ(j→i) (± 0.002)					[M+H] ^+^
				αPr_Ln	Ln_L	L_O	O_P	P_S	
1	αPr _3_	6.814	0.276						867.3
2	αPr _2_Ln	7.788	0.362	0.086					869.5
3	αPrLn _2_	8.956	0.446	0.085					871.5
4	αPr _2_L	9.511	0.481		0.120				871.8
5	αPrLnL	11.041	0.566	0.084	0.119				873.1
6	αPr _2_O	12.191	0.620			0.138			873.4
7	αPrL _2_	13.754	0.684		0.118				875.4
8	αPrLnO	14.257	0.703	0.083		0.137			875.5
9	αPrLnP	15.252	0.737				0.035		850.5
10	αPrLO	17.956	0.820		0.118				877.7
11	αPrLP	19.293	0.856		0.118		0.036		852.4
12	αPrLnS	19.718	0.867					0.129	877.7
13	αPrO _2_	23.693	0.956			0.136			879.2
14	αPrLS	25.121	0.984		0.118				879.4
15	αPrOP	25.531	0.992			0.136	0.036		883.4
Mean value				0.084	0.118	0.136	0.035	0.129	

* The peak number was listed in Figure 8.

## Figures and Tables

**Table 1:** Chromatography parameters and TAG components of *I. walleriana* seed oil.

No	TAGs	t_R_(min)	logk	Increments Δ(j→i) (± 0.002)				
				X_Ln	Ln_L	L_O	O_P	P_S
1	X_3_*	7.710	0.140					
2	X_2_Ln	8.637	0.222	0.082				
3	XLn_2_	9.755	0.303	0.082				
4	X_2_L	10.100	0.326		0.104			
5	Ln_3_	11.135	0.387	0.083				
6	XLnL	11.523	0.408	0.082	0.104			
7	X_2_O	12.270	0.445			0.119		
8	Ln_2_L	13.281	0.491	0.084	0.104			
9	XL_2_	13.762	0.512		0.104			
10	XLnO	14.136	0.527	0.082		0.119		
11	XLnP	14.828	0.553				0.027	
12	LnL_2_	15.953	0.594		0.102			
13	Ln_2_O	16.404	0.609	0.082		0.118		
14	XLO	17.038	0.629		0.103			
15	XLP	17.928	0.656		0.103		0.027	
16	XLnS	18.232	0.665					0.112
17	LnLO	19.890	0.711	0.082				
18	XO_2_	21.317	0.747			0.117		
19	XLS	22.237	0.768		0.103			0.112
20	XOP	22.500	0.774			0.118	0.028	
21	L_2_O	24.390	0.815		0.104			
22	LnO_2_	25.160	0.830	0.084		0.119		
23	LnOP	26.490	0.856				0.026	
Mean value				0.083	0.103	0.118	0.027	0.112
**linseed**								

* X- conjugated octadecatetraenoic moiety

**Table 2:** Chromatography and mass spectra parameters and TAG components of seed oils *I. walleriana*.

No*	TAGs	t_R_(min)	logk	Increments Δ(j→i) (± 0.002)					[M+H]^+^
				αPr_Ln	Ln_L	L_O	O_P	P_S	
1	αPr_3_	8.429	0.410						867.3
2	αPr_2_Ln	9.775	0.497	0.087					869.5
3	αPrLn_2_	11.413	0.584	0.087					871.5
4	αPr_2_L	12.244	0.622		0.125				871.8
6	αPrLnL	14.416	0.708	0.086	0.124				873.1
7	αPr_2_O	16.134	0.766			0.144			873.4
9	αPrL_2_	18.369	0.831		0.123				875.4
10	αPrLnO	19.126	0.852	0.085		0.143			875.5
11	αPrLnP	20.509	0.886				0.034		850.5
14	αPrLO	24.619	0.975		0.123				877.7
15	αPrLP	26.485	1.010		0.124		0.035		852.4
16	αPrLnS	27.237	1.023					0.137	877.7
18	αPrO_2_	33.282	1.117			0.143			879.2
19	αPrLS	35.364	1.146		0.123			0.136	879.4
20	αPrOP	35.986	1.154			0.144	0.036		883.4
Mean value				0.087	0.124	0.144	0.035	0.136	

* The peak number was listed in Figure 4.

**Table 3:** The composition of the main TAGs of two species *Impatiens* seed oils.

TAGs	Mole fraction of TAGs, % (±0.12, n = 3)			
	*Impatiens balsamina L*.		*Impatiens walleriana Hook.f*.	
	RID detector	UV detector	RID detector	UV detector
Pr_3_*	0.43	0.27	0.63	0.23
**Pr** **_2_** **Ln**	**18.23**	**19.17**	**12.03**	**16.71**
**PrLn** **_2_**	**9.11**	**13.18**	**11.91**	**14.49**
**Pr** **_2_** **L**	**13.31**	**11.64**	**8.65**	**9.93**
Ln_3_	1.47	-**	1.54	-
**PrLnL**	**8.17**	**10.00**	**11.17**	**11.6**
**Pr** **_2_** **O**	**7.41**	**6.79**	**6.58**	**6.62**
Ln_2_L	0.65	-	1.29	-
PrL_2_	1.79	2.10	2.94	2.30
**PrLnO**	**7.17**	**8.97**	**10.71**	**9.34**
PrLnP	5.28	7.71	3.34	7.14
LnL_2_	1.06	-	1.64	-
Ln_2_O	0.70	-	1.07	-
PrLO	4.23	4.60	1.96	4.54
PrLP	3.57	2.64	2.78	2.95
PrLnS	3.57	5.49	1.69	5.92
LnLO	1.15	-	1.10	-
PrO_2_	3.78	3.85	2.39	4.09
PrLS	2.20	1.63	2.02	1.83
PrOP	2.25	1.96	2.87	2.34
L_2_O	0.89	-	1.93	-
LnO_2_	0.90	-	6.85	-
LnOP	2.66	-	2.92	-

* Pr – parinaric moiety, including α and β parinaric.

** - not detected.

**Table 4:** Impatiens oil composition calculations for UV and RF detection after corrections.

Fatty acid	Mole fraction of acids, %				
	*Impatiens balsamina*			*Impatiens walleriana*	
	RF	UV	[19]	RF	UV
(Pr) C18:4	43.45 ± 0.87	46.05 ± 0.14	27.51	36.72 ± 0.70	44.58 ± 0.18
(Ln) C18:3	24.50 ± 0.43	25.90 ± 0.09	27.90	28.20 ± 0.33	26.55 ± 0.13
(L) C18:2	13.59 ± 0.25	11.57 ± 0.15	16.59	14.00 ± 0.19	11.80 ± 0.14
(O) C18:1	11.94 ± 0.31	10.00 ± 0.08	15.78	15.87 ± 0.22	10.34 ± 0.11
(P) C16:0	4.59 ± 0.13	4.10 ± 0.05	7.36	3.97 ± 0.14	4.15 ± 0.08
(S) C 18:0	1.92 ± 0.11	2.37 ± 0.06	4.06	1.24 ± 0.10	2.58 ± 0.05
