# Supplementary figures and images for: Determination of triacylglycerol and fatty acid compositions of Impatiens seed oils using reverse phase high performance liquid chromatography
Source: Turk J Chem. 2022 Jun 13;46(4):1332–44. doi: 10.55730/1300-0527.3440 (PMC10395786; doi:10.55730/1300-0527.3440)

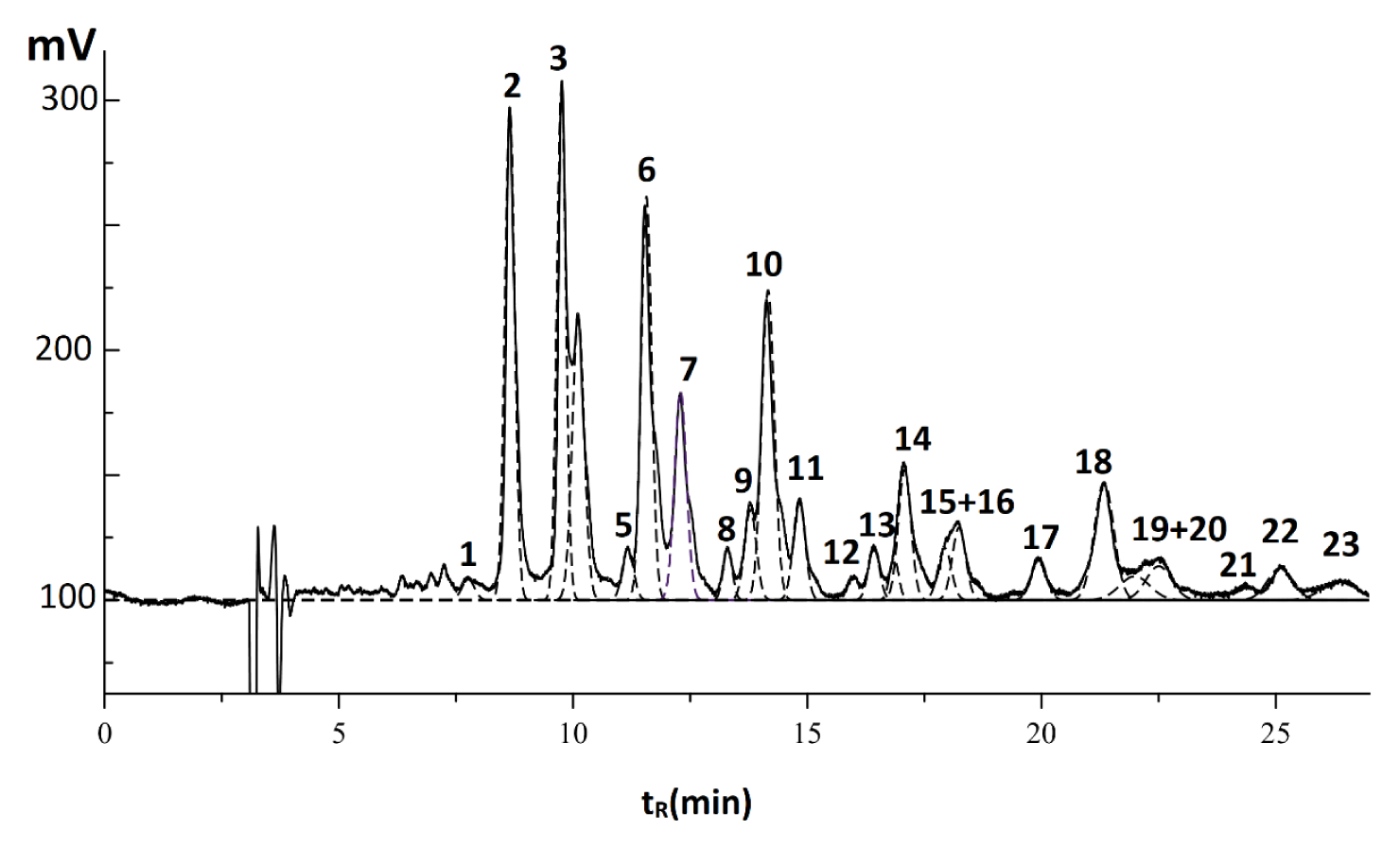

Supplement: Figure 1S — Chromatograms processed by MagicPlot Student of Impatiens walleriana; Column 4.6× 250 mm Kromasil 100× 5C18, mobile phase compositions: acetonitrile: acetone (3:7, v/v), 0.8 mL/min, refractometric index detector. [file turkjchem-46-4-1332s1.tif]
